# Supplementary material for: Attachment performance of the ectoparasitic seal louse Echinophthirius horridus
Source: Commun Biol. 2024 Jan 5;7:36. doi: 10.1038/s42003-023-05722-0 (PMC10770372; doi:10.1038/s42003-023-05722-0)
Supplement: Supplementary file 2 — Supplementary Material [file 42003_2023_5722_MOESM2_ESM.pdf]

Supplementary Data

Supplementary Data 1) List of coordinates of collection points of *E. horridus* in the North and Baltic Sea between May and November 2022.

Supplementary Data 2) Dataset of the measured attachment forces, masses, safety factors and experimental settings.

Supplementary Data 3) Table with all publications and data used for the creation of Figure 5 based on literature for interlocking in insects.

Supplementary Data 4) R scripts used for statistical tests and graphs.

Supplementary Data 5) Table of parameters for the estimation of the drag force a single seal louse, *E. horridus,* is exposed on the surface of a swimming seal.

Symbol	Parameter	Value	Unit
v	Swimming speed (seal)	4.9	m/s
S	Flow resisting area (*E. horridus*)	1.17809E-06	m^2
C_d	Drag coefficient (sphere)	0.0024	
p	Fluid density (water)	1000	kg/m^3
D	Drag force	0.03394	mN
F	Attachment force (*E. horridus*)	60.23	mN
	Attachment force/drag force	1774.60224	

The attachment force of *E. horridus* on seal fur is 1775 times stronger than the drag force generated at the most exposed area of the seal at a swimming speed of 4.9 m/s.

Supplementary Video

Supplementary Video 1) Exemplary video of a force measurement showing the detachment of *E. horridus* from seal fur.

Supplementary Video 2) Exemplary video showing the movement of *E. horridus* on seal fur and the clamping of claws on hairs.
