# Supplementary material for: Attachment performance of the ectoparasitic seal louse Echinophthirius horridus
Source: Commun Biol. 2024 Jan 5;7:36. doi: 10.1038/s42003-023-05722-0 (PMC10770372; doi:10.1038/s42003-023-05722-0)
Supplement: Supplementary file 3 — Description of Supplementary Materials [file 42003_2023_5722_MOESM3_ESM.pdf]

Description of Additional Supplementary Files

File name: Supplementary Data 1

Description: List of coordinates of collection points of *E. horridus* in the North and Baltic Sea between May and November 2022.

File name: Supplementary Data 2

Description: Dataset of the measured attachment forces, masses, safety factors and experimental settings.

File name: Supplementary Data 3

Description: Table with all publications and data used for the creation of Figure 5 based on literature for interlocking in insects.

File name: Supplementary Data 4

Description: R scripts used for statistical tests and graphs.

File name: Supplementary Data 5

Description: Table of parameters for the estimation of the drag force a single seal louse, *E. horridus*, is exposed on the surface of a swimming seal.

File name: Supplementary Video 1

Description: Exemplary video of a force measurement showing the detachment of *E. horridus* from seal fur.

File name: Supplementary Video 2

Description: Exemplary video showing the movement of *E. horridus* on seal fur and the clamping of claws on hairs.
