# Supplementary material for: Attachment performance of the ectoparasitic seal louse Echinophthirius horridus
Source: Commun Biol. 2024 Jan 5;7:36. doi: 10.1038/s42003-023-05722-0 (PMC10770372; doi:10.1038/s42003-023-05722-0)
Supplement: Supplementary file 7 — Supplementary Data 4 [file 42003_2023_5722_MOESM7_ESM.pdf]

```
##########################################################################################

This R-script is part of the manuscript "Attachment performance of the
ectoparasitic seal louse Echinophthirius horridus"

+++++++++ Boxplot Force in mN +++++++++++++

##########################################################################################


# Clear workspace
rm(list = ls())


library(ggplot2)


# Import dataset
library(readxl)
S3_Attachment_force <- read_excel("E:/Manuskripte/Attachment Force/Alles/
Supplements/S3_ Attachment_force.xlsx",
                                  sheet = "Mean_values_Boxplots")
View(S3_Attachment_force)



attach(S3_Attachment_force)


# Make boxplot

pd = position_dodge(width = 1.1)

jitter <- position_jitter(width = 0.15, height = 0.15)

p <- ggplot(S3_Attachment_force, aes(x=x_axis,
y=Mean_Corrected_values_in_mN)) +
  stat_boxplot(geom='errorbar', position = pd, width=0.1) +
  geom_boxplot(width = 0.3, position=position_dodge(width = 1.1)) +
  geom_point(position = jitter) +
  stat_summary(fun = mean, geom = "point", color = "firebrick", shape = 17,
size = 2, position = position_dodge(width = 1.1)) +
  scale_fill_manual(values=c("#999999", "#E69F00")) +
  theme_classic() +
  labs(y = "Force in mN") +
  theme(plot.title = element_text(hjust = 0.5, size = 16, face = "bold"),
plot.caption = element_text(hjust = 0, size = 10), plot.tag =
element_text(size = 16, face = "bold")) # adjust title and caption position
p
```

```
#######################################################################################

This R-script is part of the manuscript "Attachment performance of the
ectoparasitic seal louse Echinophthirius horridus"

+++++++++ Boxplot Safety Factor +++++++++++++

#######################################################################################

# Clear workspace
rm(list = ls())


library(ggplot2)


# Import dataset

library(readxl)
S3_Attachment_force <- read_excel("E:/Manuskripte/Attachment Force/Alles/
Supplements/S3_ Attachment_force.xlsx",
                                   sheet = "Mean_values_Boxplots")
View(S3_Attachment_force)



attach(S3_Attachment_force)




# Make plot

pd = position_dodge(width = 1.1)

jitter <- position_jitter(width = 0.15, height = 0.15)

p <- ggplot(S3_Attachment_force, aes(x = x_axis, y=Mean_SF)) +
  stat_boxplot(geom='errorbar', position = pd, width=0.1) +
  geom_boxplot(width = 0.3, position=position_dodge(width = 1.1)) +
  geom_point(position = jitter) +
  stat_summary(fun = mean, geom = "point", color = "firebrick", shape = 17,
size = 2, position = position_dodge(width = 1.1)) +
  scale_fill_manual(values=c("#999999", "#E69F00")) +
  theme_classic() +
  labs(y = "Safety Factor") +
  theme(plot.title = element_text(hjust = 0.5, size = 16, face = "bold"),
plot.caption = element_text(hjust = 0, size = 10), plot.tag =
element_text(size = 16, face = "bold")) # adjust title and caption position
p
```

```
#########################################################################################

This R-script is part of the manuscript "Attachment performance of the
ectoparasitic seal louse Echinophthirius horridus"

+++++++++ Kruskal-Wallis-ANOVA on ranks daily performance +++++++++++++

#########################################################################################


# Clear workspace
rm(list = ls())


library(ggplot2)
library(rstatix)
library(reshape)
library(tidyverse)
library(dplyr)
library(ggpubr)
library(plyr)
library(datarium)
library(tibble)


# Load dataset
library(readxl)
S3_Attachment_force <- read_excel("E:/Manuskripte/Attachment Force/Alles/
Supplements/S3_ Attachment_force.xlsx",
                                  sheet =
"Mean_values_dailyperformance_st")
View(S3_Attachment_force)

attach(S3_Attachment_force)


# Data preparation
S3_Attachment_force <- S3_Attachment_force %>%
  gather(key = "time", value = "Mean_SF", Day1, Day2, Day3, Day4, Day5,
Day6, Day7) %>%
  convert_as_factor(Species_shortcut, time)
data.frame(head(S3_Attachment_force, 7))


# Summary statistics
S3_Attachment_force %>%
  group_by(time) %>%
  get_summary_stats(Mean_SF, type = "mean_sd")


# Check for outliers
S3_Attachment_force %>%
  group_by(time) %>%
  identify_outliers(Mean_SF)
```

```
# Normality assumption
S3_Attachment_force %>%
  group_by(time) %>%
  shapiro_test(Mean_SF)



# Kruskal-Wallis ANOVA on ranks
res.kruskal <- S3_Attachment_force %>% kruskal_test(Mean_SF ~ time)
res.kruskal



# Due to significant difference: Post-hoc-test with Dunn's (Multiple
pairwise comparisons)
pwc2 <- S3_Attachment_force %>%
  dunn_test(Mean_SF ~ time, p.adjust.method = "bonferroni")
pwc2

print(as_tibble(pwc2), n=50)
```

```
###############################################################################################

This R-script is part of the manuscript "Attachment performance of the
ectoparasitic seal louse Echinophthirius horridus"

+++++++++ Lineplot daily performance +++++++++++++

###############################################################################################

# Clear workspace
rm(list = ls())

library(ggplot2)

# Import dataset

library(readxl)
S3_Attachment_force <- read_excel("E:/Manuskripte/Attachment Force/Alles/
Supplements/S3_ Attachment_force.xlsx",
                                  sheet = "Mean_values_dailyperformance")
View(S3_Attachment_force)


attach(S3_Attachment_force)


# Make boxplot

S3_Attachment_force$Experimental_day <-
factor(S3_Attachment_force$Experimental_day, levels=c("1", "2", "3",
"4","5", "6", "7", "8", "9", "10", "11", "12"))

pd = position_dodge(width = 1.1)

gplt <- ggplot(S3_Attachment_force, aes(x = Experimental_day, y = Mean_SF))
+
  stat_boxplot(geom='errorbar', position = pd, width=0.2) +
  geom_boxplot() +
  theme_classic()
gplt


# Add median line to boxplot

gplt +
  stat_summary(fun = median,
               geom = "line",
               aes(group = 1),
               col = "red")
```

```
##########################################################################################

This R-script is part of the manuscript "Attachment performance of the
ectoparasitic seal louse Echinophthirius horridus"

+++++++++ Mann-Whithney-U-test on sexes +++++++++++++

##########################################################################################

# Clear workspace
rm(list = ls())


library(ggplot2)
library(rstatix)
library(reshape)
library(tidyverse)
library(dplyr)
library(ggpubr)
library(plyr)
library(datarium)
library(tibble)

# Import dataset
library(readxl)
S3_Attachment_force <- read_excel("E:/Manuskripte/Attachment Force/Alles/
Supplements/S3_ Attachment_force.xlsx",
                                  sheet = "Whole_Dataset")
View(S3_Attachment_force)

attach(S3_Attachment_force)


# Mann-Whitney Test

wilcox.test(SF~Sex, data = S3_Attachment_force, exact = FALSE, correct =
FALSE, conf.int = FALSE)
```

```
##########################################################################################

This R-script is part of the manuscript "Attachment performance of the
ectoparasitic seal louse Echinophthirius horridus"

+++++++++ Shapiro-test female +++++++++++++

##########################################################################################

# Clear workspace
rm(list = ls())


library(ggplot2)
library(rstatix)
library(reshape)
library(tidyverse)
library(dplyr)
library(ggpubr)
library(plyr)
library(datarium)
library(tibble)

# Import dataset
library(readxl)
S3_Attachment_force <- read_excel("E:/Manuskripte/Attachment Force/Alles/
Supplements/S3_ Attachment_force.xlsx",
                                   sheet = "female_shapiro")
View(S3_Attachment_force)

attach(S3_Attachment_force)


# Normality assumption
shapiro.test(S3_Attachment_force$SF)
```

```
################################################################################################

This R-script is part of the manuscript "Attachment performance of the
ectoparasitic seal louse Echinophthirius horridus"

+++++++++ Linear regression weight to force +++++++++++++

################################################################################################


# Clear workspace
rm(list = ls())


library(ggplot2)
library(dplyr)
library(broom)
library(ggpubr)


# Import dataset

library(readxl)
S3_Attachment_force <- read_excel("E:/Manuskripte/Attachment Force/Alles/
Supplements/S3_ Attachment_force.xlsx",
                                  sheet = "Mean_values_Boxplots")
View(S3_Attachment_force)


attach(S3_Attachment_force)


# Check for normality

hist(S3_Attachment_force$Mean_Corrected_values_in_mN)
hist(Attachment_force$Weight_in_mg)



# Check linearity

plot(Mean_Corrected_values_in_mN ~ Weight_in_mg, data =
S3_Attachment_force)



# Linear regression analysis

weight.mean_force.lm <- lm(log_Mean_Corrected_Values ~ log_Weight, data =
S3_Attachment_force)

summary(weight.mean_force.lm)
```

```
###########################################################################################

This R-script is part of the manuscript "Attachment performance of the
ectoparasitic seal louse Echinophthirius horridus"

+++++++++ Scatterplot comparison of safety factors +++++++++++++

###########################################################################################

# Clear workspace
rm(list = ls())

library(ggplot2)

# Import dataset
library(readxl)
S6_Comparison_of_safety_factors <- read_excel("E:/Manuskripte/Attachment
Force/Alles/Supplements/S6_Comparison_of_safety_factors.xlsx",
                                              sheet = "Comparison_of_SF")
View(S6_Comparison_of_safety_factors)


attach(S6_Comparison_of_safety_factors)


# Make scatterplot

ggplot(S6_Comparison_of_safety_factors, aes(x=Shortcut_Species, y=SF)) +
  geom_point() +
  scale_y_log10() +
  theme_classic()
```

```
#####################################################################################

This R-script is part of the manuscript "Attachment performance of the
ectoparasitic seal louse Echinophthirius horridus"

+++++++++ Shapiro-test male +++++++++++++

#####################################################################################


### One-way measures ANOVA

# Clear workspace
rm(list = ls())


library(ggplot2)
library(rstatix)
library(reshape)
library(tidyverse)
library(dplyr)
library(ggpubr)
library(plyr)
library(datarium)
library(tibble)

# Import dataset
library(readxl)
S3_Attachment_force <- read_excel("E:/Manuskripte/Attachment Force/Alles/
Supplements/S3_ Attachment_force.xlsx",
                                  sheet = "male_shapiro")
View(S3_Attachment_force)

attach(S3_Attachment_force)


# Normality assumption
shapiro.test(S3_Attachment_force$SF)
```
