# Supplementary material for: Attachment performance of the ectoparasitic seal louse Echinophthirius horridus
Source: Commun Biol. 2024 Jan 5;7:36. doi: 10.1038/s42003-023-05722-0 (PMC10770372; doi:10.1038/s42003-023-05722-0)
Supplement: Supplementary file 8 — Supplementary Data 5 [file 42003_2023_5722_MOESM8_ESM.pdf]

Supplementary Data 5

Supplementary Data 5) Table of parameters for the estimation of the drag force a single seal louse, *E. horridus*, is exposed on the surface of a swimming seal.

Symbol	Parameter	Value	Unit
v	Swimming speed (seal)	4.9	m/s
S	Flow resisting area (*E. horridus*)	1.17809E-06	m^2
C_d	Drag coefficient (sphere)	0.0024	
p	Fluid density (water)	1000	kg/m^3
D	Drag force	0.03394	mN
F	Attachment force (*E. horridus*)	60.23	mN
	Attachment force/drag force	1774.60224	

The attachment force of *E. horridus* on seal fur is 1775 times stronger than the drag force generated at the most exposed area of the seal at a swimming speed of 4.9 m/s.
