# Supplementary material for: Attachment performance of the ectoparasitic seal louse Echinophthirius horridus
Source: Commun Biol. 2024 Jan 5;7:36. doi: 10.1038/s42003-023-05722-0 (PMC10770372; doi:10.1038/s42003-023-05722-0)
Supplement: Supplementary file 11 — Reporting Summary [file 42003_2023_5722_MOESM11_ESM.pdf]

Corresponding author(s): Anika Preuss, apreuss@zoologie.uni-kiel.de

Last updated by author(s): Sep 11, 2023

Reporting Summary

Nature Portfolio wishes to improve the reproducibility of the work that we publish. This form provides structure for consistency and transparency in reporting. For further information on Nature Portfolio policies, see our Editorial Policies and the Editorial Policy Checklist.

Statistics

For all statistical analyses, confirm that the following items are present in the figure legend, table legend, main text, or Methods section.

n/a	Confirmed	
☐	☒	The exact sample size (n) for each experimental group/condition, given as a discrete number and unit of measurement
☐	☒	A statement on whether measurements were taken from distinct samples or whether the same sample was measured repeatedly
☐	☒	The statistical test(s) used AND whether they are one- or two-sided<br>*Only common tests should be described solely by name; describe more complex techniques in the Methods section.*
☐	☒	A description of all covariates tested
☐	☒	A description of any assumptions or corrections, such as tests of normality and adjustment for multiple comparisons
☐	☒	A full description of the statistical parameters including central tendency (e.g. means) or other basic estimates (e.g. regression coefficient) AND variation (e.g. standard deviation) or associated estimates of uncertainty (e.g. confidence intervals)
☐	☒	For null hypothesis testing, the test statistic (e.g. F, t, r) with confidence intervals, effect sizes, degrees of freedom and P value noted<br>*Give P values as exact values whenever suitable.*
☒	☐	For Bayesian analysis, information on the choice of priors and Markov chain Monte Carlo settings
☒	☐	For hierarchical and complex designs, identification of the appropriate level for tests and full reporting of outcomes
☒	☐	Estimates of effect sizes (e.g. Cohen's d, Pearson's r), indicating how they were calculated

Our web collection on statistics for biologists contains articles on many of the points above.

Software and code

Policy information about availability of computer code

Data collection	ZEN 2008 software; AcqKnowledge software 3.7.0
Data analysis	Adobe Photoshop CS6; Affinity Photo 1.10.6; SigmaPlot 12.0; R studio 4.2.1

For manuscripts utilizing custom algorithms or software that are central to the research but not yet described in published literature, software must be made available to editors and reviewers. We strongly encourage code deposition in a community repository (e.g. GitHub). See the Nature Portfolio guidelines for submitting code & software for further information.

Data

Policy information about availability of data

All manuscripts must include a data availability statement. This statement should provide the following information, where applicable:

- Accession codes, unique identifiers, or web links for publicly available datasets
- A description of any restrictions on data availability
- For clinical datasets or third party data, please ensure that the statement adheres to our policy

All data is provided in the supplementary material of the manuscript.

Human research participants

Policy information about studies involving human research participants and Sex and Gender in Research.

Reporting on sex and gender	*Use the terms sex (biological attribute) and gender (shaped by social and cultural circumstances) carefully in order to avoid confusing both terms. Indicate if findings apply to only one sex or gender; describe whether sex and gender were considered in study design whether sex and/or gender was determined based on self-reporting or assigned and methods used. Provide in the source data disaggregated sex and gender data where this information has been collected, and consent has been obtained for sharing of individual-level data; provide overall numbers in this Reporting Summary. Please state if this information has not been collected. Report sex- and gender-based analyses where performed, justify reasons for lack of sex- and gender-based analysis.*
Population characteristics	*Describe the covariate-relevant population characteristics of the human research participants (e.g. age, genotypic information, past and current diagnosis and treatment categories). If you filled out the behavioural & social sciences study design questions and have nothing to add here, write "See above."*
Recruitment	*Describe how participants were recruited. Outline any potential self-selection bias or other biases that may be present and how these are likely to impact results.*
Ethics oversight	*Identify the organization(s) that approved the study protocol.*

Note that full information on the approval of the study protocol must also be provided in the manuscript.

Field-specific reporting

Please select the one below that is the best fit for your research. If you are not sure, read the appropriate sections before making your selection.

☒ Life sciences ☐ Behavioural & social sciences ☐ Ecological, evolutionary & environmental sciences

For a reference copy of the document with all sections, see nature.com/documents/nr-reporting-summary-flat.pdf

Life sciences study design

All studies must disclose on these points even when the disclosure is negative.

Sample size	21 adult living seal lice were used for the study; they were measured 3 times a day until their natural death occurred. 5 specimens in ethanol were used for morphological descriptions.
Data exclusions	No data was excluded from the study.
Replication	Seal lice were measured 3 times a day until their natural death occurred and only the maximum mean value (of the daily 3 measurements) of all measured days from each individual specimen was taken into account for statics and the figures.
Randomization	The testing series was randomized by measuring 21 specimens one after the other in a series determined by a dice roll.
Blinding	Blinding was not necessary as absolute values were measured and we did not directly compare values between specimens or treatments.

Reporting for specific materials, systems and methods

We require information from authors about some types of materials, experimental systems and methods used in many studies. Here, indicate whether each material, system or method listed is relevant to your study. If you are not sure if a list item applies to your research, read the appropriate section before selecting a response.

Materials & experimental systems

n/a	Involved in the study	
☒	☐	Antibodies
☒	☐	Eukaryotic cell lines
☒	☐	Palaeontology and archaeology
☐	☒	Animals and other organisms
☒	☐	Clinical data
☒	☐	Dual use research of concern

Methods

n/a	Involved in the study	
☒	☐	ChIP-seq
☒	☐	Flow cytometry
☒	☐	MRI-based neuroimaging

Animals and other research organisms

Policy information about studies involving animals; ARRIVE guidelines recommended for reporting animal research, and Sex and Gender in Research

Laboratory animals	*For laboratory animals, report species, strain and age OR state that the study did not involve laboratory animals.*
Wild animals	Adult seal lice (Echinophthirius horridus; Anoplura; Insecta) were collected during necropsies of harbour seals (Phoca vitulina) and grey seals (Halichoerus grypus), which was performed at the "Institut für Terrestrische und Aquatische Wildtierforschung" (ITAW), Büsum, Germany. The seals were found freshly dead or moribund along the North and Baltic Sea coast of Schleswig Holstein, Germany between May and November 2022. All lice investigated in the context of this study originated from seals examined in the frame of monitoring programs within the stranding network of Schleswig-Holstein to investigate their health status.
Reporting on sex	Differences between sex were tested based on a Kruskal-Wallis-Test but were not significant.
Field-collected samples	Seal lice were stored in a fridge at 4-8°C in single plastic beakers equipped with paper towels wettened in North or Baltic Sea water, respectively. Samples for morphological studies were stored in 70% ethanol.
Ethics oversight	Ethical review and approval will not be required for this study because all host animals in our study were found dead, died naturally or were euthanized based on welfare grounds and none of the host animals was killed for the purpose of this study.

Note that full information on the approval of the study protocol must also be provided in the manuscript.
